# Histopathological factors help to predict lymph node metastases more efficiently than extra-nodal recurrences in submucosa invading pT1 colorectal cancer

**DOI:** 10.1038/s41598-019-44894-w

**Published:** 2019-06-06

**Authors:** Fanny Barel, Mélanie Cariou, Philippe Saliou, Tiphaine Kermarrec, Anaïs Auffret, Laura Samaison, Amélie Bourhis, Bogdan Badic, Julien Jézéquel, Franck Cholet, Jean-Pierre Bail, Pascale Marcorelles, Jean-Baptiste Nousbaum, Michel Robaszkiewicz, Laurent Doucet, Arnaud Uguen

**Affiliations:** 1 CHRU Brest, Service d’anatomie et cytologie pathologiques, Brest, F-29200 France; 2 Registre des cancers digestifs du Finistère, Brest, F-29200 France; 3 EA7479 SPURBO, Université de Bretagne Occidentale, Brest, F-29200 France; 4 CHRU Brest, Service d’Hygiène Hospitalière- Santé Publique, Brest, F-29200 France; 5 CHRU Brest, Service d’Hépato-gastro-entérologie, Brest, F-29200 France; 6 Laboratoire d’Anatomie et Cytologie Pathologique O’Micro, Quimper, F-29000 France; 7 CHRU Brest, Service de chirurgie viscérale, Brest, F-29220 France; 8 Inserm U1053 BaRITOn, Bordeaux, F-33076 France

**Keywords:** Colonoscopy, Colorectal cancer

## Abstract

The therapeutic management of patients with endoscopic resection of colorectal cancer invading the *submucosa* (i.e. pT1 CRC) depends on the balance between the risk of cancer relapse and the risk of surgery-related morbidity and mortality. The aim of our study was to report on the histopathological risk factors predicting lymph node metastases and recurrences in an exhaustive case series comprising every pT1 CRC (of adenocarcinoma subtype only) diagnosed in Finistère (France) during 5-years. For 312 patients with at least 46 months follow-up included in the digestive cancers registry database, histopathological factors required for risk stratification in pT1 CRC were reviewed. Patients were treated by endoscopic resection only (51 cases), surgery only (138 cases), endoscopic resection followed by surgery (102 cases) or transanal resection (21 cases). Lymph node metastases were diagnosed in 19 patients whereas 15 patients had an extra-nodal recurrence (7 local recurrences only, 4 distant metastases only and 4 combining local and distant recurrences). Four patients with distant metastases died of their cancer. Poor tumor differentiation, vascular invasion and high grade tumor budding on HES slides were notably identified as strong risk-factors of lymph node metastases but the prediction of extra-nodal recurrences (local, distant and sometimes fatal) was less obvious, albeit it was more frequent in patients treated by transanal resection than with other treatment strategies. Beyond good performances in predicting lymph node metastases and guiding therapeutic decision in patients with pT1 CRC, our study points that extra-nodal recurrence of cancer is more difficult to predict and requires further investigations.

## Introduction

Colorectal cancer (CRC) is the third most common cancer in men and women worldwide, with 40,500 new cases diagnosed in France in 2011^1^. Early colorectal cancer is defined as adenocarcinoma not invading beyond the *submucosa*. It includes pTis (intraepithelial carcinoma or invading the *lamina propria*) and pT1 tumors (invading the *submucosaS*). The development of screening strategies and endoscopic treatments such as endoscopic submucosal dissection or endoscopic submucosal resection has led to an increased number of early CRC, diagnosed approximately in 0.2% to 2% of colorectal polyps removed endoscopically^2,3^. The patients treated with local endoscopic excision have a real risk of recurrence because lymph nodes metastases (LNM) are found in 6–16% of patients with pT1 CRC^4–11^. To minimize the risk of local recurrence, colorectal surgery has been recommended in Japanese and European guidelines to treat pT1 CRC diminishing the recurrence rate to 1.3% after radical surgery^12–14^. Nevertheless, a complementary colorectal surgery is not devoid of risks in terms of morbidity and mortality.

To guide treatment choices, it is necessary to identify risk factors associated with cancer recurrence and LNM. According to the guidelines of the Japanese Society for Cancer of the Colon and Rectum (JSCCR), endoscopy-resected pT1 CRC with well/moderate adenocarcinoma, no vascular invasion, submucosal invasion depth <1000 µm, grade 1 tumor budding and complete resection, have a very low risk of recurrence and metastatic evolution, and a complementary surgery would not be necessary. At the opposite, the risk of cancer recurrence and metastases is not negligible in pT1 CRC lacking one or more of the above-mentioned criteria and surgery must be discussed^12^. The decision-making process in case of pT1 CRC is complex and must be discussed at a multidisciplinary team meeting on the basis of the histopathological criteria, risks of mortality and morbidity and the informed choice of the patient.

Beside a vast majority of studies performed in East-Asia, the aim of our study was to retrospectively identify the parameters associated with cancer recurrence and metastatic evolution in a cohort of European patients with pT1 CRC diagnosed and treated using different strategies in our regional area (Finistère, France).

## Material and Methods

### Cases selection

The cases included in this study were every pT1 CRC (of adenocarcinoma subtype only) diagnosed from January the 1^st^, 2009 to December the 31^st^, 2013 in the area of Finistère (France, population 899,870 in 2011, 10 public/private hospitals with multidisciplinary and CRC-dedicated teams and networks, with CRC incidence and mortality similar to the rest of France according to 2007 to 2016 data). All cases were recorded in the Finistère Digestive Cancers Registry (FDCR) database. The FDCR records all digestive cancers in the area since 1984. The quality and exhaustiveness of the registry are certified every 4 years by an audit of the French National Committee of Registries. The FDCR collects information on patients demographics, predisposing diseases, diagnostic procedures, tumor features and stages, treatments as well as recurrences and survival. Histology slides were reviewed by two gastrointestinal pathologists (FB and LD). Cases that did not finally strictly fit in the definition of pT1 CRC of adenocarcinoma subtype were excluded. Patients with neoadjuvant treatments were also excluded. The purpose of our study was to focus on the histopathological factors associated with the risk of cancer recurrence in a comprehensive and “real life” case series of patients with pT1 CRC. We did not aim to investigate for the underlying predisposing factors such as familial cancer syndromes or inflammatory bowel diseases involved in the onset of the CRC itself. For this reason, patients were included in the study no matter they had (or not) any known cancer-predisposing factors. As a retrospective and non interventional one, the present study based on the data from the FDCR did not require informed consent of the patients and it was conducted in accordance with our national and institutional guidelines. All samples were included in a registered tumor tissue collection and the present study was conducted in accordance with the Declaration of Helsinki and after approval by our institutional review board (CHRU Brest, CPP n° DC – 2008–214).

### Histopathological analyses

A consensus was obtained between the two pathologists in the interpretation of the histopathological criteria required for the risk stratification on the basis of Hematoxylin-Eosin-Saffron (HES) slides review. The two pathologists were trained in digestive pathology and were used to the analysis of pT1 CRC slides according to the French national guidelines^15^. We assessed the histopathologic findings according to the JSCCR criteria^12^. Micrometric calibrated eyepieces were used for the measurements.

The method by Ueno *et al*. was used for the measurements of submucosal invasion depth and width^16^. The depth of the tumor submucosal invasion was measured from the superficial aspect to the deepest part of the invasion when the *muscularis mucosae* could not be identified. When the *muscularis mucosae* could be identified, it was used as the upper yardstick of the submucosal layer. The width was a measurement of the invasive front^16,17^. For pedicle polyps, Haggitt’s classification was also used scoring the depth of invasion from level 1 to level 4: level 1 in cases with invasive carcinoma limited to the head of the polyp, level 2 in cases with carcinoma invading the neck of the polyp, level 3 in cases with carcinoma invading the stalk of the polyp and level 4 in cases with carcinoma invading into the *submucosa* below the stalk of the polyp^18^. According to Haggitt’s system, we defined the “low risk Haggitt’s group” for Haggitt’s levels 1 and 2, and the “high risk Haggitt’s group” for Haggitt ‘s levels 3 and 4. For sessile tumors, a staging system adapted from the three levels of Kikuchi’s system (SM1, SM2, SM3) through the classification of Paris (i.e. SM1 corresponding to submucosal invasion <1000 µm) was used to classify the depth of submucosal invasion as SM1 (<1000 µm), SM2 (1000–2000 µm) or SM3 (>2000 µm) levels to permit the analysis of endoscopic resection samples that often do not display a *muscularis propria* required for the application of the staging system by Kikuchi^19–21^. SM1 cases were classified in ‘’low risk SM group” and SM2 and SM3 cases in ‘’high risk SM group”. Each pathologist decided which cases were suitable for Haggitt’s or SM1-SM3 classifications.

Each pathologist also decided whether the measurement of the vertical and lateral margins could be assessed or not. For the resection margin status, when assessable, R0 and R1 statuses were concluded respectively if the tumor was located more than 1 mm (R0) or 1 mm or less (R1) from the resection margin^22,23^. The presence of intra-epithelial neoplasia on the lateral margins was also noted.

For the grade of differentiation, we used the 4 grades classification given by the World Health Organization: grade 1 for well-differentiated adenocarcinoma with more than 95% gland formation, grade 2 for moderately differentiated adenocarcinoma with 50–95% gland formation, grade 3 for poorly differentiated adenocarcinoma with less than 50% gland formation and grade 4 for undifferentiated carcinoma lacking any gland formation or mucin production. Mucinous, signet-ring cells and micropapillary adenocarcinomas were individualized when the percentages of the corresponding tumor components were greater than 50%^24^. “High grade” tumors included the poorly differentiated adenocarcinoma, signet ring cells carcinoma, micropapillary and undifferentiated tumors.

Lymphatic invasion was diagnosed in case of cancer cells seen within endothelial cell-lined small vessels and venous invasion when tumor cells were seen in the lumen of large vessels with a muscle layer. Tumor perineural infiltration was also noted as present or absent.

Finally, the tumor budding was quantified following the recommendations for reporting tumor budding of the International Tumor Budding Consensus Conference 2016 (ITBCC)^25^. The tumor budding was defined as isolated single cells or clusters of less than 5 cells without gland formation at the front of the tumor. Pathologists first searched for the area with the highest tumor budding at low magnification and then counted the number of buds within this “hot spot” area to finally express their density in terms of buds per 0.785 mm² surface. Tumor budding was scored as follows: grade 1 low grade in cases with 0–4 buds per 0.785 mm² surface, grade 2 medium grade in cases with 5–9 buds per 0.785 mm² surface and grade 3 high grade in cases with 10 or more buds per 0.785 mm² surface. Tumor budding was divided into low grade (including score 1) and high grade (including scores 2 and 3)^25^.

According to the JSCCR guidelines, patients with pT1 CRCs were retrospectively classified as having “low risk” tumors if all the following criteria were present: R0 margins, low grade (i.e grade 1 or 2) tumor differentiation, adenocarcinoma of no signet-ring cells or mucinous subtype, no vascular invasion, an invasion depth <1000 µm and a low grade tumor budding. Any tumor lacking any of these criteria was classified as a “high risk” tumor^12^.

### Immunohistochemistry

Immunohistochemistry (IHC) slides searching for lymphovascular invasion images (cytokeratin plus podoplanin and cytokeratin plus CD31 dual color IHC slides) and tumor budding (cytokeratin IHC slides) were also analyzed. IHC were performed using the Ventana Benchmark XT® automated slide preparation system (Ventana- Roche Diagnostics, Meylan, France) using the UltraView Universal DAB Detection Kit (Ventana- Roche Diagnostics) and the UltraView Universal Alkaline Phosphatase Red Detection Kit (Ventana- Roche Diagnostics) with the following antibodies: for cytokeratin, clone AE1/AE3 (1:50 dilution, Dako, Glosstrup, Denmark), for podoplanin, clone D2–40 (pre-diluted, Dako) and for CD31, clone IC/70 A (1:20 dilution, Dako).

### Statistical analyses

The association between clinical, pathological and evolution data were examined using univariate analyses (i.e. chi-squared test, Fisher’s exact test and analysis of variance -ANOVA- when appropriate) and multivariate logistic regression analyses. The variables to include in the logistic regression model were chosen on the basis of the factors listed in pT1 CRC-dedicated guidelines and on the basis of the results of univariate analyses. Multivariate logistic regression analyses with backward method (i.e. removing sequentially the non-significant variables) were used. Possible interactions between independent risk factors were tested by including proper cross-product terms in the best regression model, and likelihood ratio tests comparing models with and without the interaction term were used to estimate the significance of the interaction. The level of significance was set at p < 0.05. The statistical analysis was performed using the software “R” 64.3.4.0.

### Compliance with ethical standards

Data were registered in the digestive cancer registry of Finistere database certified by the French National Committee of Registries and the present study was conducted in accordance with the Declaration of Helsinki and after approval by our institutional review board with tumor samples registered in a tumor tissue collection (CHRU Brest, CPP n° DC – 2008–214).

## Results

### Cases included

Data and slides from 342 patients were collected. After data and slides review, 30 patients were excluded because of synchronous cancer (n = 2), non-adenocarcinoma histological subtype (n = 4) and re-classed pT2 (n = 6) or pTis (n = 18) CRC. A total of 312 patients were finally retained for our study consisting in 125 (40.06%) women and 187 (59.94%) men with a mean age of 68 years (±1.26 years). The rectum and the colon were involved in 92 (29.49%) cases and 220 (70.51%) cases respectively with 53 (24.09%) tumors arising in the right colon, 4 (1.82%) in the transversal colon and 162 (73.64%) in the left colon (colonic location without further detail was reported for 1 case).

Treatment strategies consisted in endoscopic resection only (ER) in 51 (16.34%) cases, surgery resection (SR) only in 138 (44.23%) cases, endoscopic resection followed by complementary surgery (ER + SR) in 102 (32.69%) cases and transanal resection (TR) in 21 (6.73%) cases.

At the time of our study, all patients were followed for at least 46 months after initial treatment or until death. During this follow-up, isolated local recurrences (i.e. recurrences within the surgical field for colon cancer, within the pelvis for rectal cancer or in areas contiguous to the primary resection site) were observed in 7 (2.24%) cases and distant metastases were diagnosed in 8 (2.56%) cases (in association with a local recurrence in 4 cases and without local recurrence in 4 cases). All distant metastases were metachronous. Among the 234 patients who underwent colorectal surgery including contributive lymph node dissection (no lymph node analyzed for 6 patients in the SR and SR + ER groups), 19 (8,12%) cases had loco-regional LNM. Four (1.28%) patients died of the metastatic evolution of CRC. Because of the small number of patients with nodal and extra-nodal recurrences, we chose to consider in a single group the colonic and rectal tumors to perform the statistical analyses.

### Histopathological data

The mean tumor size (greater diameter) was 22.67 mm (±1.55 mm). The polyps were pedunculated in 140 (44.87%) cases and non-pedunculated (i.e. sessile or flat) in 172 (55.13%) cases. Applying the Haggitt’s classification to the 140 pedunculated polyps, submucosal invasion was classified as level 1 in 52 (38.52%) cases, level 2 in 28 (20.74%) cases, level 3 in 53 (39.26%) cases and level 4 in 2 (1.48%) cases (level not analyzable in 5 polyps). Consequently, among pedunculated tumors, 80 (59.31%) polyps were classified in the “low risk Haggitt’s group” and 55 (40.69%) polyps in the “high risk Haggitt’s group”. Quoting the SM levels in non-pedunculated tumors, the numbers of SM1, SM2 and SM3 tumors were 66 (22.84%), 83 (28.72%) and 140 (48.44%) respectively. Consequently, 223 (77.16%) polyps were classified in the “high risk SM group” and 66 (22.84%) in the “low risk SM group”.

Among the 174 patients not treated by surgical resection only (i.e. ER, ER + SR and TR groups), vertical and lateral resection margins were positive (R1) in 55 (31.61%) cases and in 7 (4.02%) cases respectively, with margins not assessable in 9 (5.17%) and 46 (26.43%) cases respectively.

Tubulous and tubulovillous adenomas were the most common precursors within 182 (58.52%) and 99 (31.83%) cases respectively. The differentiation of the CRC was grade 1 in 118 (37.82%) cases, grade 2 in 178 (57.05%) cases and grade 3 in 16 (5.13%) cases. Therefore, 296 (94.87%) cases were classified as “low grade” and 16 (5.13%) cases as “high grade”.

Analyzing HES slides, high grade tumor budding was identified in 36 (11.65%) cases and vascular invasion in 37 (11.86%) cases. Using IHC slides, high grade tumor budding, lymphatic and venous invasions were identified in 111 (38.01%) cases, 64 (21.92%) cases and 20 (6.85%) cases respectively. Perineural invasion was detected in 6 (1.92%) cases.

### Comparisons of clinical and pathological data across different treatment strategies

The comparisons between treatment strategies are summarized in Table 1. Significant trends were noted about the lower mean age of the patients that underwent ER + SR, about the greater mean size of polyps in patients treated using TR and about a less frequent ER strategy in case of non-pedunculated polyps. Patients with R1 status of deep and lateral margins were more frequently treated by ER + SR. Lateral margins more frequently contained intra-epithelial neoplasia in the TR group. Patients treated by ER only had a depth and a width of invasion inferior to other groups, inferior SM and Haggitt’s levels and did not include any mucinous adenocarcinoma. High grade tumor budding on HES and IHC slides were more frequent in the SR group. No significant difference between the treatment strategies was observed about the histological grades, vascular invasion, perineural invasion and LNM.

**Table 1:** Summary of the cases by treatment strategies.

Factors	Treatment strategies				Total	p Value
	(SR)	(ER)	(ER + SR)	(TR)		
**Sex**						
Male	75 (54,35%)	35 (68,63%)	62 (60,78%)	15 (71,43%)	187 (59,94%)	0,2049
Female	63 (45,65%)	16 (31,37%)	40 (39,22%)	6 (28,57%)	125 (40,06%)	
Mean Age ± sd	70,16 ± 1.96	70,00 ± 3.25	64,04 ± 1.96	71,24 ± 6.39	68,21 ± 1.26	0,0001*
Mean Polyp’s size (mm)	27,80 ± 2.39	16,31 ± 2.19	16,20 ± 1.42	35,83 ± 11.33	22,67 ± 1.55	<0.0001*
**Year of Incidence**						
2009	37 (26,81%)	7 (13,73%)	17 (16,67%)	2 (9,52%)	63 (20,19%)	0,1858
2010	19 (13,77%)	11 (21,57%)	25 (24,51%)	3 (14,29%)	58 (18,59%)	
2011	20 (14,49%)	12 (23,53%)	21 (20,59%)	5 (23,81%)	58 (18,59%)	
2012	31 (22,46%)	10 (19,61%)	20 (19,61%)	3 (14,29%)	64 (20,51%)	
2013	31 (22,46%)	11 (21,57%)	19 (18,63%)	8 (38,10%)	69 (22,12%)	
**Location**						
Colon	101 (73,19%)	39 (76,47%)	80 (78,43%)	0 (0%)	220 (70,51%)	<0.0001*
Rectum	37 (26,81%)	12 (23,53%)	22 (21,57%)	21 (100%)	92 (29,49%)	
**Polyp type**						
Not Pedunculated	84 (60,87%)	17 (33,33%)	56 (54,90%)	15 (71,43%)	172 (55,13%)	0,0030*
Pedunculated	54 (39,13%)	34 (66,67%)	46 (45,10%)	6 (28,57%)	140 (44,87%)	
**Resection type①**						
Monobloc	—	47 (92,16%)	88 (86,27%)	17 (80,95%)	152 (87,36%)	0,3580
Piecemeal	—	4 (7,84%)	14 (13,73%)	4 (19,05%)	22 (12,64%)	
**Adenoma②**						
Tubulous	85 (62,04%)	31 (60,78%)	54 (52,94%)	12 (57,14%)	182 (58,52%)	0,7493
Villous	8 (5,84%)	5 (9,80%)	8 (7,84%)	3 (14,29%)	24 (7,72%)	
Tubulovillous	41 (29,93%)	15 (29,41%)	37 (36,27%)	6 (28,57%)	99 (31,83%)	
Festonned	3 (2,19%)	0 (0%)	3 (2,94%)	0 (0%)	6 (1,93%)	
**Deep margin③**						
R0	138 (100%)	40 (81,63%)	50 (52,08%)	20 (100%)	248 (81,85%)	<0.0001*
R1	0 (0%)	9 (18,37%)	46 (47,92%)	0 (0%)	55 (18,15%)	
**Electrocoagulation area④**						
Invaded	—	12 (23,53%)	41 (41,00%)	3 (14,29%)	56 (32,56%)	0,0161*
Healthy	—	39 (76,47%)	59 (59,00%)	18 (85,71%)	116 (67,44%)	
**Lateral margin⑤**						
R0	138 (100%)	38 (97,44%)	70 (92,11%)	13 (100%)	259 (97,37%)	0,0066*
R1	0 (0%)	1 (2,56%)	6 (7,89%)	0 (0%)	7 (2,63%)	
**Lateral margin dysplasia**						
Yes	0 (0%)	6 (11,76%)	9 (8,82%)	4 (19,05%)	19 (6,09%)	<0.0001*
No	138 (100%)	45 (88,24%)	93 (91,18%)	17 (80,95%)	293 (93,91%)	
**Total height⑥**						
<10 mm	109 (82,58%)	44 (93,62%)	89 (89,00%)	19 (95,00%)	261 (87,29%)	0,1696
≥10 mm	23 (17,42%)	3 (6,38%)	11 (11,00%)	1 (5,00%)	38 (12,71%)	
**Depth of invasion⑦**						
<1000 µm	22 (17,19%)	19 (38,78%)	22 (23,91%)	3 (15,00%)	66 (22,84%)	0,0226*
≥1000 µm	106 (82,81%)	30 (61,22%)	70 (76,09%)	17 (85,00%)	223 (77,16%)	
**Width of invasion⑧**						
<4000 µm	38 (29,23%)	29 (64,44%)	36 (36,73%)	7 (35,00%)	110 (37,54%)	0,0005*
≥4000 µm	92 (70,77%)	16 (35,56%)	62 (63,27%)	13 (65,00%)	183 (62,46%)	
**Haggitt’s sytem⑨**						
Low Risk (levels 1–2)	27 (50,94%)	27 (79,41%)	21 (50,00%)	5 (83,33%)	80 (59,26%)	0,0146*
High Risk (levels 3–4)	26 (49,06%)	7 (20,59%)	21 (50,00%)	1 (16,67%)	55 (40,74%)	
**SM1 to SM3 system⑩**						
Low Risk (SM1)	22 (17,19%)	19 (38,78%)	22 (23,91%)	3 (15,00%)	66 (22,84%)	0,0226*
High Risk (SM2-SM3)	106 (82,81%)	30 (61,22%)	70 (76,09%)	17 (85,00%)	223 (77,16%)	
**Mucinous adenocarcinoma**						
Yes	12 (8,70%)	0 (0%)	8 (7,84%)	2 (9,52%)	22 (7,05%)	0,0216*
No	126 (91,30%)	51 (100%)	94 (92,16%)	19 (90,48%)	290 (92,95%)	
**Vascular invasion HES**						
Yes	22 (15,94%)	7 (13,73%)	6 (5,88%)	2 (9,52%)	37 (11,86%)	0,0919
No	116 (84,06%)	44 (86,27%)	96 (94,12%)	19 (90,48%)	275 (88,14%)	
**Lymphatic invasion D2–40⑪**						
Yes	31 (24,60%)	11 (24,44%)	19 (18,81%)	3 (15,00%)	64 (21,92%)	0,6452
No	95 (75,40%)	34 (75,56%)	82 (81,19%)	17 (85,00%)	228 (78,08%)	
**Venous invasion CD31⑫**						
Yes	13 (10,24%)	2 (4,44%)	4 (3,96%)	1 (5,26%)	20 (6,85%)	0,2814
No	114 (89,76%)	43 (95,56%)	97 (96,04%)	18 (94,74%)	272 (93,15%)	
**Perineural invasion**						
Yes	3 (2,17%)	0 (0%)	1 (0,98%)	2 (9,52%)	6 (1,92%)	0,0978
No	135 (97,83%)	51 (100%)	101 (99,02%)	19 (90,48%)	306 (98,08%)	
**Differentiation**						
Low Grade (grades 1–2)	132 (95,65%)	49 (96,08%)	95 (93,14%)	20 (95,24%)	296 (94,87%)	0,8246
High Grade (grades 3–4)	6 (4,35%)	2 (3,92%)	7 (6,86%)	1 (4,76%)	16 (5,13%)	
**Tumor Budding HES⑬**						
High Grade (grade 2–3)	25 (18,25%)	5 (10,00%)	4 (3,92%)	2 (10,00%)	36 (11,65%)	0,0049*
Low Grade (grade 1)	112 (81,75%)	45 (90,00%)	98 (96,08%)	18 (90,00%)	273 (88,35%)	
**Tumor Budding IHC⑭**						
High Grade (grade 2–3)	63 (48,84%)	16 (36,36%)	25 (24,75%)	7 (38,89%)	111 (38,01%)	0,0029*
Low Grade (grade 1)	66 (51,16%)	28 (63,64%)	76 (75,25%)	11 (61,11%)	181 (61,99%)	
**Lymph Node Metastasis⑮**						
Yes	12 (8,89%)	—	7 (7,07%)	0 (0%)	19 (8,05%)	0,8388
No	123 (91,11%)	—	92 (92,93%)	2 (100%)	217 (91,95%)	
**High risk/low risk tumors**						
High risk	110 (79,71%)	36 (70,59%)	85 (83,33%)	17 (80,95%)	248 (79,49%)	0,2032
Low risk	28 (20,29%)	15 (29,41%)	17 (16,67%)	4 (19,05%)	64 (20,51%)	

Not available/mesurable results not shown in the table:

①Resection type = 138/②Adenoma = 1/③Deep margin = 9/④Electrocoagulation area = 140/⑤Lateral margin = 46/⑥Total height = 13/⑦Depth of invasion = 23/⑧Width of invasion = 19/⑨Haggitt’s system = 177/⑩ SM1-SM3 system = 23/⑪Lymphatic invasion D240 = 20/⑫ Venous invasion CD31 = 20/⑬Tumor budding HES = 3/⑭Tumor budding IHC = 20/⑮Lymph Node Metastasis = 76;

SR: surgical resection only; ER: endoscopic resection only; ER + SR: endoscopic resection followed by surgical resection; TR: transanal resection; HES: Hematoxylin-Eosini-Saffron; D2–40: podoplanin immunohistochemistry; CD31: CD31 immunohistochemistry; IHC: immunohistochemistry.

### Risk factors associated with lymph node metastases

The comparisons between patients with and without LNM among patients with SR and ER/SR based treatments are summarized in Table 2. The Table 3 summarizes the clinical and pathological data of the 19 patients with nodal metastases. In univariate analyses, the presence of vascular invasion on HES slides, perineural invasion, positive lateral margin on endoscopically-resected samples, poor tumor differentiation and high tumor budding on HES slides were significantly associated with LNM. In multivariate analysis, only the presence of vascular invasion on HES slides (Odds Ratio: 9.32, CI:2.83–31.86, p = 0.0002) and poor differentiation (Odds Ratio:16.87, CI:4.16–70.90, p < 0.0001) were independent factors associated with LNM. Every patients with LNM were classified as having high risk tumors according to the JSCCR guidelines^12^.

**Table 2:** Summary of factors associated with lymph node metastases in patients with surgical lymph node dissection.

Factors	Lymph node metastases		Total	p Value
	Present	Absent		
	n = 19	n = 215		
**Sex**				
Male	8 (42,11%)	126 (58,60%)	134 (57,26%)	0,2495
Female	11 (57,89%)	89 (41,40%)	100 (42,74%)	
Mean Age ± sd	67,58 ± 5.62	67,33 ± 1.50	67,35 ± 5.39	0,9280
Mean Polyp’s size ± sd	22,39 ± 4.60	23,17 ± 1.83	23,1 ± 1.72	0,8110
**Location**				
Colon	12 (63,16%)	165 (76,74%)	177 (75,64%)	0,2616
Rectum	7 (36,84%)	50 (23,26%)	57 (24,36%)	
Polyp type				
Pedunculated	7 (36,84%)	92 (42,79%)	99 (42,31%)	0,8093
Not Pedunculated	12 (63,16%)	123 (57,21%)	135 (57,69%)	
**Resection type①**				
Monobloc	4 (57,14%)	81 (88,04%)	85 (85,86%)	0,0567
Piecemeal	3 (42,86%)	11 (11,96%)	14 (14,14%)	
**Deep margin②**				
R0	13 (68,42%)	172 (82,30%)	185 (81,14%)	0,2148
R1	6 (31,58%)	37 (17,70%)	43 (18,86%)	
**Electrocoagulation area on endoscopic pieces③**				
Invaded	5 (71,43%)	33 (36,67%)	38 (39,18%)	0,1577
Healthy	2 (28,57%)	57 (63,33%)	59 (60,82%)	
**Lateral margin④**				
R0	14 (87,50%)	189 (98,44%)	203 (97,60%)	0,0485*
R1	2 (12,50%)	3 (1,56%)	5 (2,40%)	
**Lateral margin dysplasia**				
Yes	0 (0%)	9 (4,19%)	9 (3,85%)	1,0000
No	19 (100%)	206 (95,81%)	225 (96,15%)	
**Residual disease on surgical pieces⑤**				
Yes	1 (14,29%)	2 (2,17%)	3 (3,03%)	0,1994
No	6 (85,71%)	90 (97,83%)	96 (96,97%)	
**Surgery type**				
Abdomino-perineal amputation	5 (26,32%)	43 (20,00%)	48 (20,51%)	0,2837
Segmentary colectomy.	12 (63,16%)	162 (75,35%)	174 (74,36%)	
Total colectomy	2 (10,53%)	9 (4,19%)	11 (4,70%)	
Hartmann’s	0 (0%)	1 (0,47%)	1 (0,43%)	
**Number of ganglions analyzed⑥**				
<12	8 (44,44%)	103 (50,24%)	111 (49,78%)	0,8212
≥12	10 (55,56%)	102 (49,76%)	112 (50,22%)	
**Lymph node metastases⑦**				
1	12 (63,16%)	—	12 (63,16%)	—
2	5 (26,32%)	—	5 (26,32%)	
3	1 (5,26%)	—	1 (5,26%)	
4	1 (5,26%)	—	1 (5,26%)	
**Adenoma component⑧**				
Villous	0 (0%)	16 (7,48%)	16 (6,87%)	0,3746
Festonned/Tubulous/Tubulo-villous	19 (100%)	198 (92,52%)	217 (93,13%)	
**Total height⑨**				
<10	15 (78,95%)	178 (85,99%)	193 (85,40%)	0,4927
≥10	4 (21,05%)	29 (14,01%)	33 (14,60%)	
**Depth of SM invasion(µm)⑩**				
<1000	1 (5,88%)	42 (21,21%)	43 (20,00%)	0,2050
≥1000	16 (94,12%)	156 (78,79%)	172 (80,00%)	
**Width of SM invasion(µm)⑪**				
<4000	7 (36,84%)	66 (32,51%)	73 (32,88%)	0,8975
≥4000	12 (63,16%)	137 (67,49%)	149 (67,12%)	
**Haggitt’s system⑫**				
Low Risk (levels 1–2)	1 (14,29%)	47 (54,02%)	48 (51,06%)	0,0564
High Risk (levels 3–4)	6 (85,71%)	40 (45,98%)	46 (48,94%)	
**SM1 to SM3 system⑬**				
Low Risk (SM1)	1 (5,88%)	42 (21,21%)	43 (20,00%)	0,2050
High Risk (SM2-SM3)	16 (94,12%)	156 (78,79%)	172 (80,00%)	
**Mucinous adenocarcinoma**				
Yes	0 (0%)	20 (9,30%)	20 (8,55%)	0,3830
No	19 (100%)	195 (90,70%)	214 (91,45%)	
**Vascular invasion HES**				
Yes	8 (42,11%)	19 (8,84%)	27 (11,54%)	0,0004*
No	11 (57,89%)	196 (91,16%)	207 (88,46%)	
**Lymphatic invasion D2–40**				
Yes	7 (41,18%)	42 (20,59%)	49 (22,17%)	0,0663
No	10 (58,82%)	162 (79,41%)	172 (77,83%)	
**Venous invasion CD31**				
Yes	3 (16,67%)	14 (6,86%)	17 (7,66%)	0,1477
No	15 (83,33%)	190 (93,14%)	205 (92,34%)	
**Perineural invasion**				
Yes	2 (10,53%)	2 (0,93%)	4 (1,71%)	0,0341*
No	17 (89,47%)	213 (99,07%)	230 (98,29%)	
**Differentiation**				
Low Grade (grades 1–2)	13 (68,42%)	210 (96,77%)	223 (94,49%)	0,0002*
High Grade (grades 3–4)	6 (31,58%)	7 (3,23%)	13 (5,51%)	
**Tumor Budding HES**				
High Grade (grade 2–3)	6 (31,58%)	22 (10,28%)	28 (12,02%)	0,0157*
Low Grade (grade 1)	13 (68,42%)	192 (89,72%)	205 (87,98%)	
**Tumor Budding IHC**				
High Grade (grade 2–3)	11 (57,89%)	74 (36,10%)	85 (37,95%)	0,1039
Low Grade (grade 1)	8 (42,11%)	131 (63,90%)	139 (62,05%)	

Not Applicable results not shown in the statistic table:

①Resection Type = 135/②Deep Margin = 6/③Electrocoagulation Area = 137/④Lateral Margin = 26/⑤Tumor Remaining = 135/⑥Number of Ganglions taken = 11/⑦Lymph Node Metastasis = 215/⑧Adenoma component = 1/⑨Total height = 8/⑩Depth of invasion = 19 /⑪Width of invasion = 12/⑫Haggitt’system = 140/⑬SM1 to SM3 system = 19.

**Table 3:** Clinical and pathological features of the 19 patients with lymph node metastases.

No.	Age	Sex	Location	SM depth (µm)	SM width (µm)	SR vs ER + SR	Margins	Resection method	Differentiation	V	P	Tumor budding	Number of nodes retrieved	Number of LNM	Therapy for LNM
1	64	Female	Left colon	5750	11500	SR	−	N/A	Low	−	−	Low	6	1	Chemotherapy
2	65	Male	Rectum	3950	7250	SR	−	N/A	Low	+	+	High	—	1	Chemotherapy
3	80	Female	Rectum	1400	21500	SR	−	N/A	Low	+	−	Low	8	1	—
4	62	Female	Left colon	2075	2750	ER + SR	+	En bloc	Low	−	−	Low	15	2	Chemotherapy
5	47	Female	Rectum	1398	3500	ER + SR	+	En bloc	High	−	−	Low	9	2	Chemotherapy
6	75	Female	Right colon	2950	6000	ER+SR	+	Piecemeal	Low	−	−	High	14	1	—
7	69	Male	Rectum	—	5500	SR	−	N/A	Low	−	−	Low	6	4	Chemotherapy
8	82	Female	Left colon	1950	1400	SR	−	N/A	Low	+	−	High	12	2	Chemotherapy
9	62	Male	Left colon	—	9500	ER+SR	+	En bloc	Low	−	−	Low	16	2	Chemotherapy
10	60	Female	Left colon	483	2615	SR	−	N/A	Low	+	−	Low	12	1	Chemotherapy
11	69	Female	Left colon	2350	4500	ER+SR	−	Piecemeal	Low	+	−	Low	12	1	Chemotherapy
12	71	Female	Right colon	2198	7000	SR	−	N/A	High	+	+	High	18	2	Chemotherapy
13	50	Female	Left colon	3250	7000	SR	−	N/A	Low	+	−	High	13	1	Chemotherapy
14	85	Male	Rectum	2040	8500	SR	−	N/A	Low	−	−	Low	7	3	—
15	65	Male	Right colon	1850	2300	ER+SR	+	Piecemeal	High	−	−	Low	16	1	Chemotherapy
16	74	Female	Left colon	5315	12500	SR	−	N/A	High	−	−	Low	13	1	Chemotherapy
17	45	Male	Rectum	2320	1000	ER+SR	+	En bloc	Low	−	−	Low	3	1	Chemotherapy
18	80	Male	Left colon	1695	2050	SR	−	N/A	High	+	−	Low	3	1	Chemotherapy
19	79	Male	Rectum	4130	11500	SR	−	N/A	High	−	−	High	4	1	Chemotherapy

SM = submucosal; SR = surgery resection; ER + SR = Endoscopy resection followed by surgery; Margins = horizontal and vertical margins of endoscopy resection; Low grade differentiation = grade 1–2, High grade differentiation = grade 3–4, V = vascular invasion, P = perineural invasion, Low tumor budding = grade 1, High tumor budding = grade 2–3, LNM = Lymph Node Metastases.

### Risk factors associated with extra-nodal local and distant recurrences

**Figure 1:**
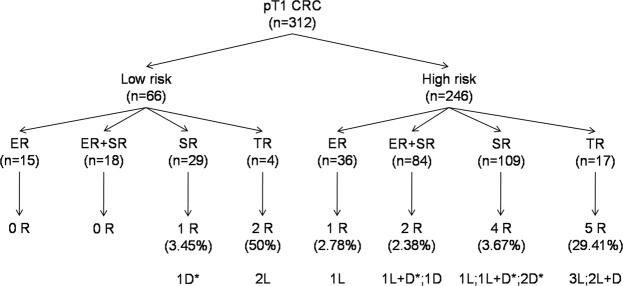
Flow diagram summarizing the patients with local and distant recurrences and cancer-related deaths by histopathological risk factors and treatment strategies. CRC: colorectal cancer; ER: endoscopic resection only; ER + SR: endoscopic resection followed by surgical resection; SR: surgical resection only; TR: transanal resection; R: recurrence; D: distant recurrence; L: local recurrence; L + D: local and distant recurrence; *patients with cancer-related deaths.

Among the 15 patients with local recurrences, 7 patients had isolated local recurrences, 4 patients had local recurrences associated with distant metastases and 4 patients had distant metastases without local recurrence. Only 7 of the 15 patients had a surgical treatment including lymph node dissection and none had any loco-regional LNM, including the 4 patients who died from the metastatic evolution of CRC. The Table 4 summarizes the clinical and pathological data of the 15 patients with local/distant recurrences. Twelve among 15 (80%) patients with local recurrences and/or distant metastases were classified as having high risk tumors according to the JSCCR guidelines^12^. The risk factors associated with local recurrences and distant metastases could not be investigated separately because of the small number of patients having these different (and overlapping for 4 patients) relapse profiles with different treatment strategies. Of note, we observed high proportions of recurrences in the group of patients treated by TR (2/4 patients with low risk tumors and 5/17 patients with high risk tumors, see Fig. 1 for details).

**Table 4:** Clinical and pathological features of the 15 patients with extra-nodal recurrences.

No.	Age	Sex	Location	SM depth (µm)	SM width (µm)	Margins	Resection method	Diff.	V	P	Tumor budding	LNM	Months to recurrence	Recurrence site	Alive/Dead
***ER*** = ***Endoscopy resection***															
1	81	Male	Left colon	NE	NE	+	En bloc	Low	+	−	NE	NA	52	local	Non cancer death
***TR*** = ***Transanal resection***															
2	70	Male	Rectum	NE	NE	−	Piecemeal	Low	−	−	NE	NA	26	local	Alive
3	84	Male	Rectum	3065	15000	−	En bloc	Low	−	+	Low	NA	28	local	Non cancer death
4	75	Male	Rectum	2515	6500	−	En bloc	Low	+	−	High	NA	25	local	Non cancer death
5	58	Male	Rectum	985	2150	−	En bloc	Low	−	−	Low	NA	7	local	Alive
6	56	Male	Rectum	2830	8500	−	En bloc	Low	−	−	Low	NA	22	local + distance(liver)	Non cancer death
7	71	Male	Rectum	1700	1560	NA	Piecemeal	Low	−	−	Low	NA	30	local	Alive
8	48	Female	Rectum	2000	11500	−	En bloc	Low	−	−	High	0/12	40	local+distance(peritoneal)	Alive
***ER***+***SR*** = ***Endoscopic resection followed by surgical resection***															
9	62	Male	Left colon	1825	2100	−	En bloc	Low	NA	−	Low	0/11	49	local + distance(lung + others)	Cancer Death
10	51	Male	Left colon	2050	2000	−	En bloc	Low	−	−	Low	0/12	53	distance(lung)	Alive
**SR** = **Surgical resection**															
11	68	Male	Left colon	4190	11000	−	−	Low	+	−	High	0/12	34	local	Non cancer death
12	74	Female	Rectum	1970	4000	−	−	Low	−	−	High	NA	34	local + distance(lung)	Cancer death
13	69	Male	Right colon	4473	11000	−	−	Low	−	−	Low	0/13	43	distance(lung)	Alive
14	82	Female	Right colon	315	1650	−	−	Low	+	−	High	0/11	27	distance(liver+others)	Cancer death
15	75	Female	Rectum	900	2000	−	−	Low	−	−	Low	0/12	55	distance(peritoneal)	Cancer death

SM = submucosal; Margins = horizontal and vertical margins of endoscopy resection; Diff: differenciation Low (grae 1–2) vs High (grade 3–4) grade differentiation;V = vascular invasion, P = perineural invasion, Low tumor budding grade = grade 1, High tumor budding grade = grade 2 3, LNM = Lymph Node Metastasis. NA: not assessable.

## Discussion

The advances in endoscopic diagnosis and resection methods have resulted in an increased detection of early CRC. pT1 CRC are often treated by local endoscopic resection followed (or not) by complementary surgery. They rarely relapse but a minority of pT1 CRCs can metastasize to the regional lymph nodes or even distantly to other organs, as seen in our study. It is necessary to better determine the factors predicting the risk of recurrence and metastatic evolution of pT1 CRC for appropriate therapeutic choices in patients with high or low risk tumors.

LNM occur in 6–16% of pT1 CRCs (8.12% in our study)^4–11^. The risk factors taken into account in the JSCCR guidelines for discussing a complementary surgery only in case of a “high grade tumor” appeared also relevant in our series because every patient with LNM were classified as having a high risk tumor^12^. Among these risk factors, also now listed in the standardized pathological report recommended by the French Society of Pathology, poor differentiation, high grade tumor budding and vascular invasion on HES slides particularly emerged as strong predictors of LNM^15^. Albeit being an important predictive factor in pT1 CRC using different methodologies in the literature, the depth of tumor invasion was not correlated with any recurrence or metastatic evolution in our series^15,17,26–29^. Moreover, some of these methodologies are difficult to apply in endoscopic, e.g. the SM1-SM3 Kikuchi’s system inapplicable in pT1 CRC samples lacking a deep band of *muscularis propria*. As previously evocated by some authors, the depth of invasion could be a weaker predictive value than other risk factors in pT1 CRC^7^. A major risk factor of metastatic evolution is a poor differentiation^2,26,30,31^. It represents between 2.4% and 7.2% of pT1 CRC (5.51% of cases in our series)^3,16,32–34^. Another major risk factor of metastases is vascular invasion, in blood or in lymphatic vessels^26,27,34–37^. Albeit the diagnosis of vascular invasion could be challenging on HES sections and that IHC analyses using endothelial markers (e.g. podoplanin and CD31) could be helpful in this diagnosis, beyond the confirmation of an ambiguous image on HES section, the real prognostic interest of IHC remains uncertain for risk stratification in pT1 CRC^26,35,37^. The same controversial interest of IHC in comparison with HES exists about the diagnosis and grading of tumor budding. Emphasizing the guidelines of the ITBCC 2016, a HES-based diagnosis appeared adequate for the prediction of metastatic evolution with no need of IHC-based analysis^25^. Albeit not mentioned as a major risk factor in the JSCCR guidelines, perineural invasion was associated with metastatic evolution in our study and would merit to be taken into account for discussing therapeutic decisions in patients with pT1 CRC^38^.

Even after surgical resection with lymph nodes dissection, about 2% of pT1 CRCs developed local and/or metastatic recurrences during a median follow-up period of 7.8 years^39^. The local and/or metastatic recurrence rates after endoscopic resection were reported to be very low (0–2.3%) in the absence of risk factors for LNM^40–43^. Patients with pT1 CRC treated using TR are reported to have higher rates of recurrence (between 2% and 24%)^44,45^. Our results are in accordance with the recurrence rates reported in the literature pointing out notably higher recurrence rates using TR than with ER, SR or ER + SR. Nevertheless, as almost constantly reported in studies searching for parameters associated with extra-nodal recurrences in pT1 CRC, the small number of patients with various recurrence types prevented us to draw formal conclusions about the prognostic value of the different histopathological parameters among the various types of recurrences, i.e. local or/and distant ones. This small number also prevented us to perform separate analyses of colonic tumors and rectal tumors. In the current literature, rectal location, poor differentiation and vascular invasion are reported to be more frequent in patients with extra-nodal recurrences in general^39,41,42,46,47^. Searching for the factors associated with local or distant recurrences would merit to be the subject of additional studies in the future. According to the current guidelines, additional surgery is also strongly recommended if endoscopically-resected pT1 CRC show positive margins because of a high risk of residual tumor and recurrence. However, during the process of endoscopic resection, some tissue of submucosal safe margin might be lost by electrical cauterization and, beside a R1 margin on the pathological sample, the resection could have been curative enough to prevent a local recurrence. This could at least partially explain why a positive margin did not appear as a risk factor for recurrence in our study.

Positive lymph node status was reported to be the only significant independent predictor of 5-year cancer specific survival and 5-year disease-free survival in patients with pT1 and pT2 colorectal cancers^8^. In our study, the 19 patients diagnosed with LNM had no recurrence after surgery, 16 of them having been treated by chemotherapy because of their pN + status. Nevertheless, 4 patients died of their cancer because of distant metastases and none had loco-regional LNM. Therefore, it is important to further identify risk factors associated with distant metastases to adapt the surveillance and therapeutic management of patients. A better understanding of these rare cases with “unexpected evolution” is important to better anticipate and avoid damageable cancer recurrences. This would require the identification of these rare cases in large cohorts, maybe in nationwide ones, with patients treated and followed up homogeneously, but also with standardized pathological reports helping to build databases of “early CRC with unexpected recurrences” that could permit to point new prognostic factors helping the clinicians in their therapeutic decisions.

## Conclusion

In our study, we confirmed the prognostic value of the histopathological parameters that must absolutely appear in a pathological report of a pT1 CRC^12,15^. We also pointed that, beyond an help in case of ambiguous image on HES slides, IHC analyses searching for vascular invasion and tumor budding were not more useful than HES for risk stratification in pT1 CRC. The histopathological risk stratification, integrated in a case-by-case discussion by a multidisciplinary team, has permitted to propose an adequate treatment strategy for patients at risk of LNM. Nevertheless, whereas colorectal surgery (as an initial treatment or as a complementary treatment after initial endoscopic resection) has permitted to diagnose LNM and to adapt the subsequent therapeutic management of the pN + patients who did not relapse, some patients with high risk tumors lacking any LNM have developed distant metastases and died of their cancer.

In other words, can we predict the recurrence of cancer in case of pT1 CRC? The answer is yes, but with imperfect predictive values. At this time, we can neither recommend more or less local resection or radical surgery in comparison with current guidelines. Additional studies remain necessary to better predict, early detect and treat local and distant recurrences in patients with a pT1 CRC history. Molecular biomarkers, liquid biopsy but also digital image analyses and artificial intelligence methods applied to endoscopic images and whole histology slides may be of interest in this field of prognostication of early CRC and will merit future works. Waiting for these potential new prognostic approaches, we strongly believe that the complexity of decision making in case of pT1 CRC merits that each case 1) should be analyzed by two pathologists to reach consensual histoprognostic data in the pathological report, 2) should be referred to a polyps-dedicated multidisciplinary team meeting for a therapeutic decision after weighting the pros and cons of performing a complementary surgery, 3) should undergo a long time and regular follow-up because recurrences can arise even a long time after the initial diagnosis and treatment in patients having low risk or high risk tumors.
